# Rising spillover risk: Lessons from the Andes hantavirus outbreak

**DOI:** 10.1016/j.onehlt.2026.101605

**Published:** 2026-10-10

**Authors:** Dechuan Kong

**Affiliations:** Shanghai Municipal Center for Disease Control and Prevention, Shanghai, China

**Keywords:** Spillover, Accelerating human–nature contact, Pandemic probability, Disease X, Andes hantavirus, Ecological surveillance

## Abstract

The unprecedented expansion of human activity boundaries is turning spillover from a chance event into a foreseeable ecological and epidemiological certainty. Using the 2026 Andes hantavirus cruise ship outbreak as an entry point, this study examines it within a broader driver framework: nearly all emerging infectious disease events in humans in recent decades have originated from wildlife transmission; under climate-change and land-use scenarios, approximately 3139 mammal species are projected to aggregate in new combinations by 2070, causing an estimated 4000 cross-species viral transmission events. The rapid expansion of tourism into ecologically fragile regions — sub-Antarctic waters, the deep sea, and primary forests — the persistence of habitat fragmentation and animal trade, and the reshaping of wildlife distribution and vector dynamics by extreme climate events together constitute “novel contact amplifiers” that accelerate contact at the human–animal–environment interface. Robotics, artificial intelligence, autonomous driving, and automated logistics are extending human activities into previously inaccessible regions, yet they remain almost entirely absent from current spillover risk assessments. The limitations of cruise ship medical systems — whose core capacity lies in “recognize, isolate, stabilize, and transfer” rather than definitive diagnosis and treatment — were fully exposed in this event. From a One Health perspective, this paper proposes that responses to spillover risk must shift from passive reaction to proactive interface governance, incorporate technology-driven increases in contact frequency into prevention and control frameworks, and advance cornerstone strategies such as broad-spectrum vaccines and broad-spectrum antiviral drugs at the basic research level.

While the recent emergence of the MV Hondius Andes orthohantavirus [1], [2], [3]is linked to a distinct spillover event from wild rodent reservoirs to humans in a previously unknown-risk area, such spillover events are not anomalous per se; it is the specific circumstances of this event that are exceptional. It is a harbinger. As anthropogenic pressure on intact ecosystems intensifies, the frequency of such spillovers will inevitably increase — not as a matter of probability, but as a matter of ecological and epidemiological certainty. In recent years, nearly all emerging infectious disease events in humans have originated from wildlife transmission [4]. In 2008, Jones, K. E., et al. analyzed a database of 335 Emerging infectious diseases (EIDs) “events” (origins of EIDs) between 1940 and 2004, demonstrating non-random global distribution patterns with a significant increase in EID events over time after controlling for reporting bias [4]. In 2022, Carlson et al. provided robust quantitative evidence for this conclusion: under climate-change and land-use scenarios, approximately 3139 mammal species will aggregate in new combinations by 2070, causing an estimated 4000 cross-species viral transmission events, with bats accounting for the majority of novel viral sharing — and holding warming under 2 °C is insufficient to reduce this trajectory [5]. The study makes clear that this ecological shift is likely already underway.

Anthropogenic pressure increases transmission risk through multiple ecological pathways by increasing contact frequency. Humanity has never been in such deep and extensive contact with nature as it is today [6], [7]. Whether it is the depths of the ocean we can now reach, the breadth of terrestrial landscapes we can traverse, or the altitudes in the sky we can attain, the footprint of human activity is penetrating every corner of the planet at an unprecedented scale. Yet what is even more critical — and more alarming — is not merely the expanding breadth and depth of these incursions, but the astonishingly accelerating rate at which these changes are occurring [8], [9]. Humanity's exponentially expanding ecological footprint is compressing the physical distance between humans and wildlife at an unprecedented frequency and intensity, thereby building a “highway” for cross-species pathogen transmission [7], [10].

Firstly, a defining characteristic of contemporary human outdoor tourism is its unprecedented expansion into previously inaccessible and ecologically fragile environments, encompassing sub-Antarctic cruises, deep-sea diving, spaceflight, and trekking through undisturbed forest ecosystems—a trend that intrinsically elevates the frequency and intimacy of human contact with wildlife reservoirs, thereby facilitating zoonotic spillover events [4]. Recent outbreaks linked to adventure travel underscore this growing threat: the 2026 Andes virus outbreak on a sub-Antarctic cruise vessel, with 13 confirmed cases and three fatalities across multiple nations, exemplifies how a single travel-acquired infection can rapidly disseminate through confined passenger settings [1], [11], [12]. This is not an isolated incident but part of a concerning pattern; increased ecotourism in Southeast Asia has been associated with potential pathogen exposure from macaque populations at popular temple sites, where non-human primates carry zoonotic agents including herpes B virus and simian foamy virus [13], [14]. Similarly, adventure tourism in cave ecosystems has been linked to histoplasmosis outbreaks due to disturbance of bat guano, with documented infection clusters following cave exploration in Brazil, Ecuador, and others [15], [16], [17]. The growth of space tourism further introduces the possibility of immunocompromised individuals in confined spacecraft encountering reactivated or novel infectious agents, as spaceflight induces immune system alterations that may increase vulnerability to opportunistic infections [18]. Collectively, these events illustrate how the modern quest for exploration, while valuable, creates dynamic and high-risk interfaces at the human-animal-environment nexus, demanding enhanced pre-travel risk assessment, real-time surveillance, and rapid response capacities to mitigate the growing global health threats posed by travel-acquired emerging diseases [19].

Secondly, the accelerated encroachment and alteration of animal habitats are intensifying human-wildlife interfaces. Habitat fragmentation forces previously isolated wildlife species into new assemblages, creating opportunities for viral transmission between hosts without prior contact history [20]. Deforestation, agricultural expansion, and rapid urbanization directly elevate zoonotic risk—particularly for mosquito-, rodent-, and bat-borne diseases—by breaking down natural ecological barriers and drastically shortening the spatial distance between humans and wildlife [21]. Moreover, habitat disturbance reshapes wildlife microbiomes, and animals living near humans may acquire both environmental and human-associated microorganisms, facilitating cross-species transmission and potential “spillback” [22], [23], [24].

Thirdly, animal trade cannot be effectively prohibited. The global legal and illegal wildlife trade represents one of the most significant pathways for the emergence of new infectious diseases, as it brings diverse species—often stressed and confined in overcrowded conditions—into close contact with each other and with humans [25]. Live animal markets, particularly in regions with high biodiversity, serve as mixing vessels where pathogens can cross species barriers, amplify, and acquire novel mutations capable of infecting human hosts [26] The trade in exotic pets, bushmeat, and traditional medicine further exacerbates this risk, with species such as non-human primates, bats, and rodents being transported across continents, carrying viruses including Ebola, Nipah, and multiple coronaviruses [27]. Moreover, the lack of adequate veterinary screening, quarantine protocols, and traceability systems means that infected animals enter supply chains undetected, allowing pathogens to spread silently across borders before any clinical case is recognized in humans [28]. Even when international bans exist, enforcement is hampered by corruption, limited resources, and the high profitability of illicit trafficking [29]. Collectively, the inability to effectively prohibit animal trade perpetuates a high-risk interface for zoonotic spillover, demanding urgent global cooperation in surveillance, market reform, and supply chain regulation to mitigate future pandemic threats [30].

In addition to anthropogenic activities, global climate change and the increasing frequency of extreme natural disasters are fundamentally reshaping the geographical distribution and behavioral patterns of wildlife, thereby accelerating the occurrence of spillover events. Rising global temperatures have driven poleward and upward shifts in species ranges, forcing previously isolated wildlife populations into novel sympatric assemblages, which creates unprecedented opportunities for cross-species pathogen exchange [5]. Extreme weather events—including floods, droughts, and heatwaves—further exacerbate this dynamic by disrupting ecological equilibria, altering vector population dynamics, and increasing vector-borne disease transmission in previously non-endemic regions [31]. For instance, El Niño-associated flooding has been linked to Orthohantavirus pulmonary syndrome outbreaks in the Americas, as heavy rains boost rodent populations and facilitate human-rodent contacts [32]. Similarly, warming temperatures have expanded the geographical range of tick species, contributing to the emergence of Lyme disease and tick-borne encephalitis in higher latitudes [33]. These climatic and disaster-driven ecological disruptions, operating synergistically with human land-use changes, are effectively rewilding pathogen landscapes.

Finally, from an epidemiological framework perspective, contact frequency and transmission probability are positively correlated. The basic reproduction number(R₀) of zoonotic diseases is directly correlated with susceptible host density and contact frequency. As population growth and urbanization proceed, human population density reaches thresholds that allow R₀ > 1, providing conditions for the establishment of novel zoonotic diseases in human populations. Cross-species transmission is fundamentally a problem of contact probability. When the ecological footprint of human activity compresses the physical distance between humans and wildlife at unprecedented frequency and intensity, the opportunities for “chance encounters” with pathogens increase exponentially.

Amid this accelerating contact, pathogenic microorganisms that naturally reside in wildlife are encountering humans by chance [7], [34]. Some of these chance encounters lead to pathogens that can transmit rapidly among humans, as seen with SARS-CoV-2. Others infect only a few individuals and then cease further human transmission — as seen in the recent Hondius Andes orthohantavirus outbreak.Yet each such encounter is like a box of chocolates in *Forrest Gump*: you never know what colour it will bring to humanity [35]. But as the frequency of human–wildlife contact increases, so too does the probability that one of these encounters will yield a pandemic with devastating consequences [26].Assessing the risk of cross-species pathogen transmission centers on judging the likelihood that a spillover event will occur. Traditional models often emphasize contact frequency alone (e.g., “humans entering wildlife habitats”), but single-driver explanations have limited explanatory power. The “eco-evolutionary alignment” framework proposed in 2026 argues that spillover risk rises disproportionately only when multiple ecological and evolutionary “pillars” converge in time and space [36]. These pillars include host network structure (the density and connectivity of human–wildlife contact), pathogen evolutionary potential (genetic diversity and recombination capacity of viruses in reservoir hosts), environmentally mediated factors (the effects of climate and land-use change on host distribution and pathogen survival), and persistence pathways (the ability of pathogens to survive in the environment and find new hosts) [36], [37], [38]. Its testable hypothesis is that if contact is frequent but evolutionary and ecological conditions are not aligned, spillover attempts remain constrained; conversely, high viral diversity in reservoir hosts without repeated ecological connectivity is also unlikely to generate sustained spillover. At the operational level, the SpillOver platform provides a quantifiable risk-ranking tool that integrates host, viral, and environmental risk factors based on expert opinion and scientific evidence, scoring 887 wildlife viruses for spillover potential. Validation shows that the top 12 are all known zoonotic viruses, and several newly discovered viruses score higher than some known pathogens, indicating that risk ranking can identify priority surveillance targets before novel pathogens infect humans [39]. The PREDICT project, from a large-scale epidemiological perspective, confirms that mammal diversity, land-use type, and land-use change are the most important predictors of emerging infectious diseases from wildlife, with the highest risk in regions of high mammal diversity [4].

Faced with such an inevitably increasing spillover risk, how should we respond? Our focus should not rest on generalities. On the one hand, we must address the gaps that the MV Hondius Orthohantavirus Outbreak has exposed as requiring urgent attention; on the other hand, we must focus on the emerging drivers that are amplifying human–wildlife contact frequency at an unprecedented speed and scale. The rapid expansion of tourism channels large populations into ecologically sensitive areas [40]. Advances in robotics and artificial intelligence extend human activities into previously inaccessible regions — such as the deep sea, polar zones, and primary forests. The proliferation of autonomous driving and automated logistics systems is reshaping human mobility patterns and spatial distribution across terrestrial landscapes. While bringing economic benefits, these technological transformations are quietly remaking the human–nature interface, acting as “novel contact amplifiers” beyond traditional ecological disruptors — yet they have remained almost entirely absent from spillover risk assessments to date [10].

How should cruise ship medical and health services be restructured to better respond to events such as the Andes Orthohantavirus incident? Some people think that cruise ship medical and health services are not designed for infectious disease outbreaks; they are meant to meet basic healthcare and sanitation needs, not for pathogen diagnosis, specialized treatment, or end-stage care. Given this rare infectious event, it was inevitable that the cruise ship failed to respond effectively. From the standpoint of risk control, a number of actionable recommendations may be proposed. In view of the ongoing and foreseeable threats posed by emerging infectious diseases, maritime medicine must place greater emphasis on infectious disease risk management, with strategic attention to both pre-embarkation screening and rapid emergency response during the voyage. Prior to departure, a critical issue is whether feasible measures exist to identify and defer boarding for passengers carrying notifiable or high-risk infectious diseases. Once at sea, the most pressing problem is likely to be the sudden scarcity of medical resources within a confined and isolated environment.

From a risk control perspective, the following concrete measures can be proposed for maritime medicine to respond to emerging infectious diseases such as Orthohantavirus. Before departure, a health declaration system matched to the itinerary's epidemiological profile should be established, requiring passengers and crew to complete targeted symptom screening 24 to 72 h before boarding, with particular attention to fever, myalgia, arthralgia, rash, and respiratory symptoms, alongside risk assessment based on exposure history in endemic areas (such as rural travel or rodent contact in South America) [41], [42]. Clear criteria for deferring boarding should also be defined, similar to the quarantinable and monitored infectious diseases used in border entry screening by various countries, with rapid testing capacity for priority pathogens deployed at embarkation points where feasible [42]. During the voyage, the greatest challenge is the scarcity of medical resources. A tiered response mechanism should be established: a single suspected case should trigger immediate isolation and remote consultation; multiple cases or a high-consequence pathogen should activate contact tracing, cohort isolation, and diversion to the nearest capable port [41]. The ship's medical center should be equipped with rapid diagnostic tests for pathogens common along the route, personal protective equipment, and supplies sufficient for 72 to 96 h of independent operation. At the same time, a satellite-based telemedicine support system should be established to maintain real-time contact with shore-based infectious disease specialists and public health authorities, and transfer agreements should be pre-arranged with ports along the route that have adequate reception capacity. Fundamentally, cruise ship medical services are not designed for infectious disease outbreaks; their core objective should be to “recognize, isolate, stabilize, and transfer,” rather than to achieve definitive diagnosis and treatment onboard— which is precisely why adequate medical services (with trained personnel) and evacuation plans should be stressed as a way forward.

From the perspective of global public health, interoperable cross-border surveillance and secure data sharing are key components in responding to spillover events of emerging infectious diseases [43], [44]. Pathogens know no borders, and a localized spillover event can spread globally within hours through modern travel networks. Therefore, national surveillance systems must achieve interoperability in data formats, reporting standards, and laboratory testing procedures, ensuring that outbreak information can be rapidly identified, promptly reported, and effectively compared [45]. At the same time, data sharing must be conducted under the premise of safeguarding privacy, national security, and intellectual property rights, establishing a trustworthy, transparent, and sustainable international cooperation mechanism [46]. Only in this way can the international community form a coordinated response at the early stage of a spillover event, avoiding the loss of prevention and control windows caused by information delays and fragmentation [47], [48]. This should also become one of the core directions for building the future global public health governance system [44].

Nowadays, the world is entering an era of rapid scientific and technological development. Scientific and technological innovation is empowering the prevention and control of emerging infectious diseases, while also bringing risks and challenges that cannot be ignored. Advances in artificial intelligence, robotics, autonomous driving, and commercial spaceflight are profoundly transforming people's lives, transportation, medical services, and research models, among many other aspects, and are also reshaping the patterns of infectious disease transmission and spread, bringing considerable uncertainty to the future prevention and control of emerging infectious diseases. Shared autonomous vehicles, as enclosed spaces, may instead become new nodes for aerosol transmission if ventilation, surface cleaning, and passenger density are not properly controlled [49]; commercial spaceflight and microgravity environments may promote increased antibiotic resistance and virulence changes in pathogens [50], [51], while planetary protection regulations for returning spacecraft and personnel still remain a regulatory gap in the commercial sector.

The Hondius Andes Orthohantavirus outbreak itself does not pose a public health risk to us — but perhaps we were just lucky this time. Whether we treat it as a call to action or as another footnote in pandemic forensics will determine how many Hondius events we will see in the coming decade. Undoubtedly, compared with the past few decades, Disease X is arriving at an accelerated pace.

## CRediT authorship contribution statement

**Dechuan Kong:** Writing – review & editing, Writing – original draft, Conceptualization.

## Funding

"Medical Innovation Research" Project (25Y32800200) under the Shanghai Municipal Commission of Science and Technology's 2025 Key Technology Research and Development Program.

## Declaration of competing interest

The authors declare that they have no known competing financial interests or personal relationships that could have appeared to influence the work reported in this paper.

## Data Availability

No data was used for the research described in the article.
